# Male stress urinary incontinence surgery in Australia: Temporal trends over two decades

**DOI:** 10.1002/bco2.70205

**Published:** 2026-04-22

**Authors:** Omattage Mahasha Perera, Yam Ting Ho, Femi E. Ayeni, Eric Chung, Vincent Tse, Devang Desai

**Affiliations:** ^1^ Department of Urology Ipswich Hospital Ipswich Queensland Australia; ^2^ Nepean Institute of Academic Surgery The University of Sydney School of Medicine Sydney New South Wales Australia; ^3^ Department of Urology Princess Alexandra Hospital, University of Queensland Woolloongabba Queensland Australia; ^4^ Department of Urology Macquarie University Hospital Sydney New South Wales Australia; ^5^ Department of Urology Concord Hospital Sydney New South Wales Australia; ^6^ Department of Urology Toowoomba Specialists East Toowoomba Queensland Australia; ^7^ Faculty of Medicine University of Queensland Brisbane Queensland Australia; ^8^ Faculty of Medicine Griffith University Gold Coast Queensland Australia; ^9^ Faculty of Medicine University of Southern Queensland Toowoomba Queensland Australia

**Keywords:** artificial urinary sphincter, national trend, retropubic, slings, stress urinary incontinence

## Abstract

**Objective:**

This study aims to evaluate the national trends in the surgical management of male stress incontinence (SUI) in the Australian population over the 21st century.

**Methods:**

Data was sourced from the Australian Institute of Health and Welfare (AIHW) national morbidity database and Medicare Benefits Schedule (MBS) statistics reports. Annual data from financial years (FY) 2000/01 to 2022/23 was collected. Male SUI procedures are grouped into slings, paraurethral bulking agents (PBA) and artificial urinary sphincters (AUS). The annual procedural counts, revisions and yearly changes were obtained. The estimated subsidy burden was reported as AUD per capita (per 100 000 persons).

**Results:**

Between FY2000/01 and 2022/23, the aggregate number of male SUI procedures (excluding revisions) increased by 272% (from 288 to 1072). This is largely driven by the increase in AUS procedures, particularly amongst men aged 70–74 years. Male sling procedures demonstrated a substantial early increase followed by plateauing in recent years, while PBA declined over the same period. Revision procedures increased by 444% (from 41 to 223), largely due to AUS revisions and replacement. Over the same period, the estimated aggregate subsidy burden has increased from $13 749 to $53 269 per capita.

**Conclusion:**

The surgical management of male SUI in Australia has been transformative over the past two decades. Trends indicate an increase in male SUI procedures, particularly AUS procedures accompanied by an increase in AUS revision and replacement. These trends reflect both expanding surgical demand and durability of continence devices, which has subsequently led to a substantial increase in estimated subsidy burden.

## 1 INTRODUCTION

Stress urinary incontinence (SUI) is a debilitating condition, which severely affects quality of life. Male SUI occurs as a result of damage to the pelvic floor structures and/or external urinary sphincter complex.^1^ This typically can occur following radical prostatectomy (RP) or transurethral resection of prostate (TURP). However, other aetiological factors can exist such as prior pelvic fractures, pelvic irradiation or neurological disorders. Despite sphincter deficiency being the most common causative factor, other bladder conditions such as poor bladder compliance or detrusor overactivity can often co‐exist and partly contribute to patient symptoms.^2^ Consequently, determining the true incidence of SUI in the male population remains challenging.

Effective SUI management requires a spectrum of interventions, ranging from conservative measures to advanced surgical techniques. Non‐invasive approaches include lifestyle modifications, pelvic floor muscle training and biofeedback therapy. Other conservative methods include the use of penile clamps or clean intermittent self‐catheterisation (CISC). Anti‐muscarinic or beta‐3 adrenergic agonist agents can be considered in certain patients exhibiting co‐existing pathologies resulting in mixed urinary incontinence.^2^ However, for SUI alone, no approved pharmacologic therapies exist. Thus, when conservative methods fail, often surgical treatments are considered.

The choice of surgical intervention is guided by patient‐specific factors, including the degree of incontinence, age, comorbidities, prior pelvic irradiation or surgical history and overall treatment expectations. For mild symptoms, minimally invasive male urethral slings can be considered in men who have not undergone pelvic radiation. Male slings can either be fixed or adjustable. Fixed male slings made of polypropylene, function by elevating the urethral bulb and applying pre‐pubic pressure. Fixed male slings include the transobturator AdVance (American Medical Systems, Minnetonka, MN, US), Virtue (Coloplast, Minneapolis, MN, USA) and I‐STOP TOMS (DiLoMedical, Lyon, France) slings.^3^ Adjustable male slings such as ProAct (Uromedica, Minnesota USA), Argus (Promedon, Cordoba, Argentina) and ATOMS (AMI, GmbH, Feldkirch, Austria) contain an adjustable mechanism to personalise the amount of compressive pressure applied on the urethra.^4^ Male slings have overall success rates ranging between 36% and 90%, with a reduction of 50% in incontinence.^2^ Other minimally invasive surgical treatments, such as paraurethral urethral bulking agents, have poor clinical evidence in terms of efficacy in male SUI and may require repeated injections with poor durability of results.^5^

Artificial urinary sphincter (AUS) is considered the gold standard treatment for men with moderate to severe SUI or stress incontinence, also when associated with radiotherapy. The AMS 800 (American Medical Systems, Minnetonka, MN US) has been in use for over 50 years and contains a circumferential urethral cuff that can be inflated and deflated by a scrotal pump. Social continence, which is the use of zero to one pads per day, is reported between 55% and 77%, with a 90% satisfaction rate at 15 years follow‐up.^6^ Due to its proven long‐term efficacy, this modality is considered the preferred therapeutic option in instances of failed male urethral sling procedures or in patients with severe baseline SUI (characterised by usage exceeding two pads per day.^7^ The Zephyr ZSI 375 (Zephyr Surgical Implants, Geneva, Switzerland) and Victo AUS (Promedon, Cordoba, Argentina) also report similarly effective clinical outcomes.^8^ Despite the promising results, as with any surgical treatments, risks and limitations exist. Rates of erosion and prosthesis associated infections are higher with AUS, with a reported explantation or revision rate of 20%–30% over time.^9^

The surgical landscape of male SUI management is a broad and complex field that requires prudent shared decision‐making and counselling dependent on patient‐specific factors. With an aging population and increasing diagnoses of prostate cancer, male SUI will continue to be a pressing yet underexplored issue in Australia. The trends in the surgical management of male SUI in Australia have not been studied to date. This study seeks to address this by evaluating the use of Medicare data to uncover patterns in procedural preferences, costs and regional disparities over the past two decades. Rising costs associated with surgical interventions, such as artificial urinary sphincter (AUS) insertion and sling procedures, underscore the need for strategic healthcare planning. This research aims to identify current trends and assess their economic impact and regional variations.

## 2 METHODS

### 2.1 Data source/collection

Data was sourced from the Australian Institute of Health and Welfare (AIHW) National Hospital Morbidity databases. These consist of health information from both public and private hospitals. Data related to procedure counts is organised under the Australian Classification of Health Interventions (ACHI)—a standardised coding for procedures and interventions in Australian hospitals based on the Medicare Benefits Schedule (MBS).

Data was collected as yearly data from financial years (FY) 2000/01 to 2022/23. FY is defined as a 12‐month period starting on 1 July and ending on 30 June of the following year. Under the ACHI, the following codes are labelled for male stress incontinence management: (i) sling procedures: 37 044; (ii) paraurethral bulking agents: 37 339; (iii) artificial urinary sphincter: 37 381, 37 384, 37 387 and 37 390. These procedure codes can be further divided into gender, age groups and revision of procedure. Description of codes can be seen in Table 1.

**TABLE 1:** The description of MBS listed codes.

MBS code	Descriptions
37044‐00	Retropubic procedure for stress incontinence: bladder stress incontinence, suprapubic operation for (such as Burch colposuspension), open or laparoscopic route, using native tissue without graft, with diagnostic cystoscopy to assess the integrity of the lower urinary tract
37044‐03	Revision of retropubic procedure for stress incontinence
37339‐01	Injection of paraurethral bulk for stress incontinence, male
37381‐00	Insertion of cuff of artificial urinary sphincter, perineal approach
37384‐00	Insertion of cuff of artificial urinary sphincter, abdominal approach
37387‐00	Insertion of artificial urinary sphincter
37390‐00	Revision of artificial urinary sphincter
37390‐01	Replacement of artificial urinary sphincter

MBS item statistics report on the rebate cost on qualified medical services provided by each state; the report is available through the Australian Government Services Australia. Qualified medical services include those provided by a registered provider and processed by Services Australia. It does not include services that qualify under the Department of Veterans Affair National treatment account. The cost is calculated by the Services Australia based on the individual benefit rate for each MBS listed procedure and the population enrolled in Medicare in that financial year. The cost is reported as Australian dollars per 100 000 persons (capita). The cost to each MBS listed procedure represents the contribution funded by the Australian government only. It does not include overall cost to the patient and cost to the healthcare system such as personnel, equipment and so forth. The cost does not differentiate between original and revision procedures.

### 2.2 Data analysis

This is a descriptive study to describe the surgical trends of SUI management, and no formal statistical testing was performed. Data was exported to a Microsoft Excel file for analysis and illustration. Procedure counts were demonstrated as aggregate and proportions. Procedural counts can be further separated by gender, age and whether a revision was performed.

## 3 RESULTS

### 3.1 Overall procedural counts

The tabulated breakdown of each procedure across all FY periods can be seen in Table 2. The year on year (YoY) change (% difference) for each procedure including revisions is noted in Table 3 and Figures S1 and S2. The aggregate number of male SUI procedures (excluding revisions) performed have increased by 272% (from 288 to 1072) between FY2000/01 and 2022/23. By proportions of SUI procedures, there was a shift in preference of procedures. For example, between FY2002/03 and 2022/23, use of AUS increased from 43% to 58%; while paraurethral injections reduced from 57% to 6%, slings have increased from 0% to 36% (Figure S3).

**TABLE 2:** Tabulated breakdown of aggregate procedural counts of MBS listed procedures, across all FY periods.

FY period	37044‐00 Retropubic procedure for stress incontinence, male	37044‐03 Revision of retropubic procedure for stress incontinence, male	37339‐01 Injection of paraurethral bulk for stress incontinence, male	37381‐00 Insertion of cuff of artificial urinary sphincter, perineal approach	37384‐00 Insertion of cuff of artificial urinary sphincter, abdominal approach	37387‐00 Insertion of artificial urinary sphincter	37390‐00 Revision of artificial urinary sphincter	37390‐01 Replacement of artificial urinary sphincter
2000/01	0	0	176	40	8	64	27	14
2001/02	0	0	184	27	12	52	32	17
2002/03	1	0	148	45	5	61	39	10
2003/04	22	1	163	43	5	62	26	11
2004/05	40	0	168	43	0	85	35	16
2005/06	64	0	192	57	0	97	38	15
2006/07	90	23	195	65	3	99	35	15
2007/08	140	33	198	63	5	129	47	16
2008/09	179	27	143	71	8	119	47	17
2009/10	279	30	104	78	7	155	54	11
2010/11	325	33	97	123	0	182	60	0
2011/12	394	42	86	152	0	238	67	0
2012/13	370	46	76	169	12	233	67	23
2013/14	380	41	61	189	15	265	70	23
2014/15	382	37	81	196	25	283	81	34
2015/16	434	71	72	263	16	360	119	55
2016/17	371	60	59	240	8	343	152	61
2017/18	337	66	109	272	17	402	117	68
2018/19	382	62	87	217	12	289	106	48
2019/20	384	42	66	263	24	303	82	66
2020/21	371	49	94	279	24	333	158	69
2021/22	342	47	80	269	18	305	121	62
2022/23	381	40	64	267	18	340	118	65

*Note*: Colour code: yellow, sling procedure; red, paraurethral injection; green, AUS.

**TABLE 3:** Tabulated breakdown of year on year change (%) of procedural counts by type of SUI and revision procedures.

	YoY (%) for procedures				YoY (%) for revisions		
FY period	A.U.S.	Injection	Retropubic	Total	A.U.S.	Retropubic	Total
2000/01	N/A	N/A	N/A	N/A	N/A	N/A	N/A
2001/02	−19	5	N/A	−5	20	N/A	20
2002/03	22	−20	N/A	−5	0	N/A	0
2003/04	−1	10	2100	13	−24	N/A	−22
2004/05	16	3	82	14	38	−100	34
2005/06	20	14	60	22	4	N/A	4
2006/07	8	2	41	10	−6	N/A	38
2007/08	18	2	56	18	26	43	32
2008/09	1	−28	28	−3	2	−18	−5
2009/10	21	−27	56	20	2	11	4
2010/11	27	−7	16	17	−8	10	−2
2011/12	28	−11	21	20	12	27	17
2012/13	6	−12	−6	−1	34	10	25
2013/14	13	−20	3	6	3	−11	−1
2014/15	7	33	1	6	24	−10	13
2015/16	27	−11	14	18	51	92	61
2016/17	−8	−18	−15	−11	22	−15	11
2017/18	17	85	−9	11	−13	10	−8
2018/19	−25	−20	13	−13	−17	−6	−14
2019/20	14	−24	1	5	−4	−32	−12
2020/21	8	42	−3	6	53	17	45
2021/22	−7	−15	−8	−8	−19	−4	−17
2022/23	6	−20	11	6	0	−15	−3

### 3.2 Types of procedures

#### 3.2.1 Male slings

**FIGURE 1:**
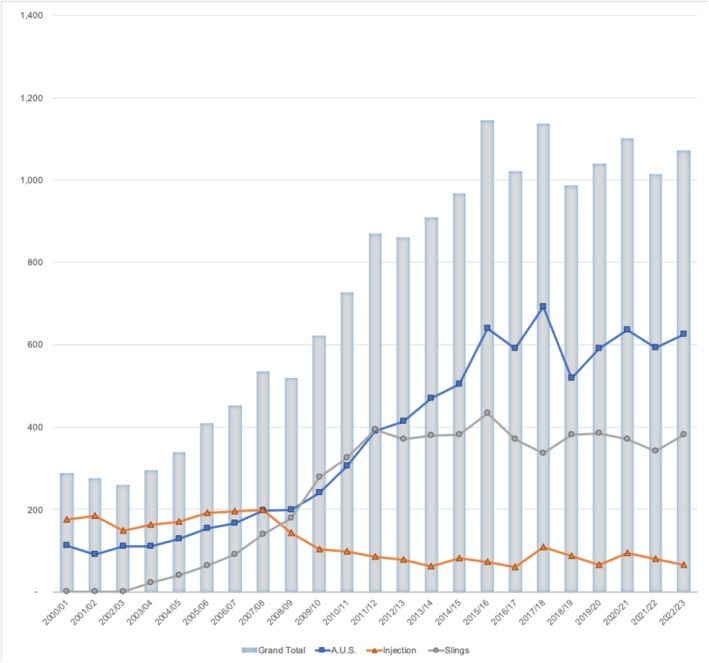
Aggregate procedural counts of all and individual types of SUI procedures (not inclusive of revision procedures) performed between FY2000/01–2022/23.

In FY2002/03, only one procedure was performed compared to 381 procedures in FY2022/23. This represents a 3800% increase. Though, observations from the trend since FY2011/12 (Figure 1) and YoY change (Table 3), the progression has plateaued, especially in the last 5 years. This procedure is commonly performed amongst patients aged 65 and 74 years old (Figure S4).

#### 3.2.2 Paraurethral bulking agents

Between FY2000/01 and 2022/23, the aggregate number of procedures decreased by 63.6%, from 176 to 64. The trend remained stable, with a slow yet steady decline during FY2008/09 (Figure 1), and this is also reflected from the YoY change in the last 5 years as well (Table 3). This procedure is commonly performed amongst those 65 and 74 years old (Figure S4).

#### 3.2.3 AUS

The aggregate procedural counts (inclusive of both AUS and cuff insertion) between FY2000/01 and 2022/23 have increased by 458%, from 112 to 625. The progression continued to show an upward trajectory since FY 2008/09, as seen on Figure 1 and Table 3. This procedure is commonly performed between patients aged 70 and 74 years (Figure S4).

### 3.3 Revision procedures

**FIGURE 2:**
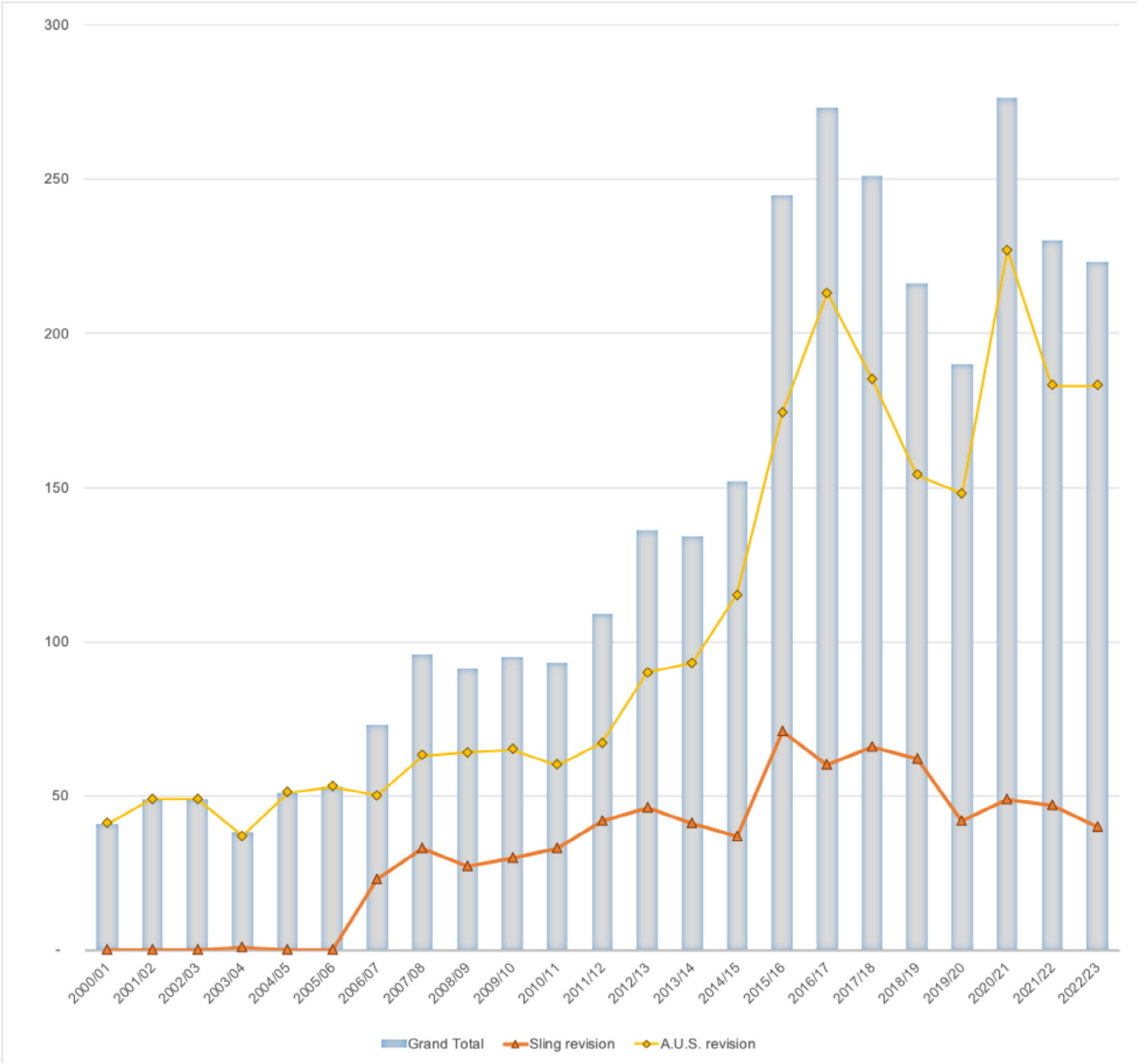
Aggregate procedural counts of all and individual types of revision procedures performed between FY2000/01–2022/23.

Between FY2000/01 and 2022/23, the aggregate number of revisions increased by 444% (from 41 to 223). Within these procedures, the most substantial disparity was observed in AUS revisions, which included both replacements and revision operations (Figure 2).

During FY 2020/21, AUS revisions reached their highest frequency with a total of 227 procedures performed. In comparison, there was one reported sling revision case between FY2000/01 and 2005/06. However, this peaked in FY2015/16, with 71 revision procedures reported. Since then, there has been a downtrend (Figure 2), which was also reflected on the YoY change (Table 3 and Figure S2). Revision procedures for AUS and sling procedures were commonly performed amongst patients aged between 70 and 74 years old (Figure S5).

### 3.4 Estimated subsidy burden

**FIGURE 3:**
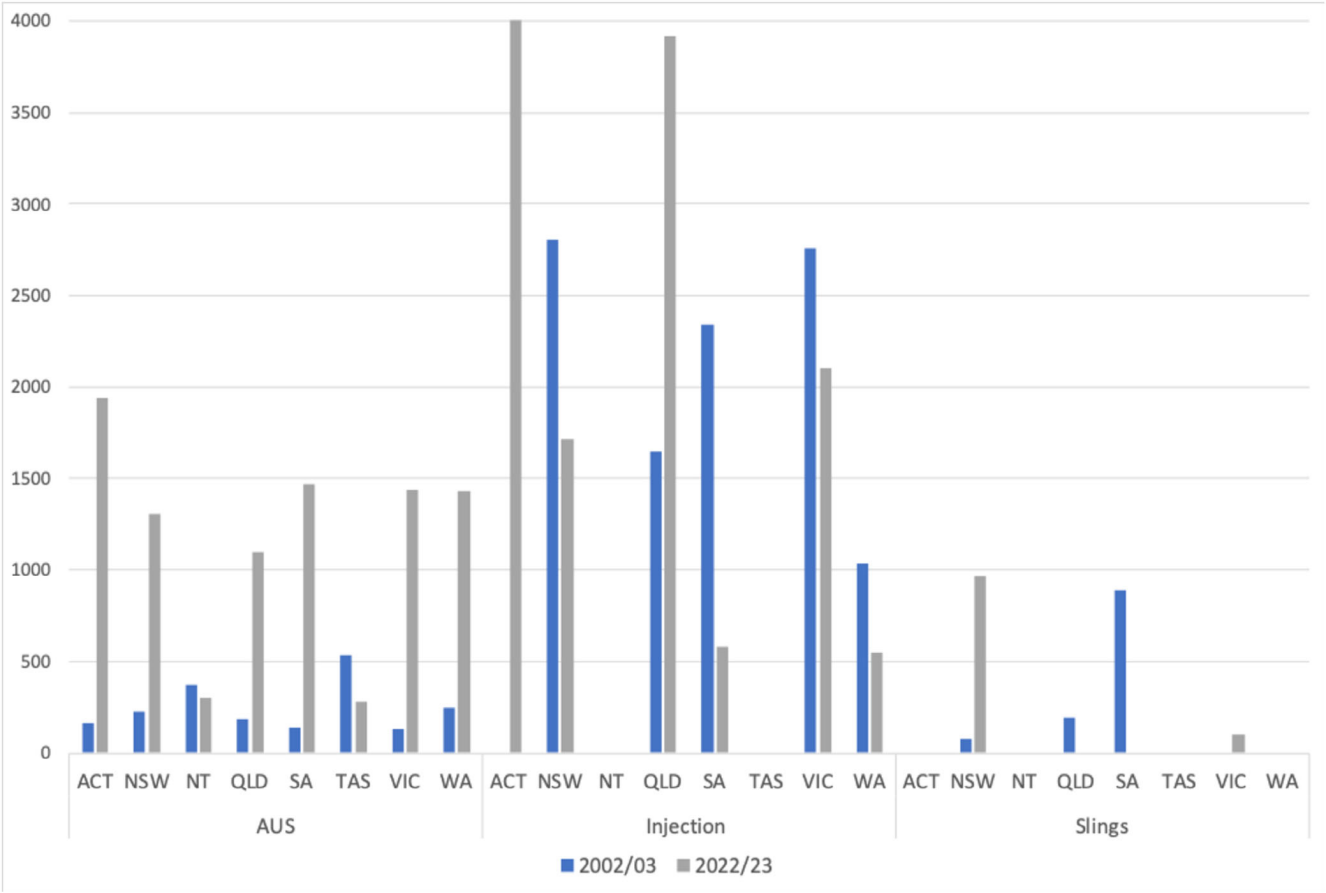
Estimated subsidy burden per capita (AUD per 100 000 persons), for all types of SUI procedures performed between FY2002/03–2022/23, across all states.

Between FY2002/03 and 2022/23, the nationwide subsidy burden for aggregate male SUI procedures demonstrated a substantial increase from $13 749 to $53 269, reflecting a 287.4% rise. Within this period, the cost increased from $1993 to $9267 per capita of AUS procedure and from $10 590 to $42 936 per capita for paraurethral injection. In contrast, sling procedures decreased from $1116 to $1066 per capita. Figure 3 illustrates state by state comparisons and AUS procedures demonstrated an increase in per capita benefit across all states, with the exception of the Northern Territory and Tasmania. Moreover, the increase in use of paraurethral injection procedures was limited to Australian Capital Territory and Queensland, whereas New South Wales contributed the largest portion of sling procedures nationwide.

## 4 DISCUSSION

The data from this study indicates a marked transformation in the surgical management of male stress incontinence management over the past two decades within Australia. Over the two decades of the 21st century, a 272.2% increase in the total number of continence procedures was observed. This rise is likely indicative of multiple contributory factors.

One such factor could be the role of the aging population demographics along with the resultant rise in uropathology as well as other medical factors which contribute to stress incontinence. According to the World Health Organization (WHO), between 2015 and 2050, it is projected that the proportion of the global population aged over 60 is projected to nearly double, increasing from 12% to 22%.^10^ With an aging population and resultantly conditions such as BPH and prostate cancer increasing in prevalence, it is expected rates of male stress urinary incontinence will increase.^11^ Roberts et al. demonstrated a 108% increase in rates of radical prostatectomy in men aged 75–84 years of age between 2002 and 2016 within Australia.^12^

Another retrospective analysis of surgical trends of BPH within Australia observed an overall progressive increase in the rate of total BPH surgical procedures (92 versus 133 cases per 100 000 men) between 2000 and 2018, with the TURP being the most commonly performed.^13^ These procedures carry a small risk but nevertheless inherent risk of incontinence. Patients who may have been continent post prostatectomy or TURP may develop intrinsic sphincteric deficiency requiring surgical management. This may also explain the increase in surgical continence management amongst the above 80‐year‐old group noted in our analysis. Alongside the ageing demographic trend, Australia has experienced a concomitant rise in metabolic syndromes and neurological disorders, which are established risk factors for male SUI.^14^ Another factor that could be contributing to the rise of continence procedures could be a shift to more minimally invasive procedures, such as slings.

Of the various interventions, male slings demonstrated the most substantial growth, from zero procedures performed in FY2002/2002, to 381 procedures performed in FY2022/23. Male urethral slings were first commercially available in 2004, following the introduction of the InVance, Argus and Remeex slings.^3^ This was shortly followed by the AdVance Sling in 2008 and the Virtue sling in 2012.^15^ The male urethral sling became a valuable addition to a urologists' armamentarium and rapidly ascended to the preferred surgical option for male SUI between 2000 and 2010. A similar exponential rise in mid‐urethral slings (MUS) was noted in females between 2003 and 2012.^16^

The marked use of male urethral slings could perhaps be a reflection of its robust efficacy in conjunction with greater surgeon familiarity.^15^ Sling insertions are generally quick procedures with a lower learning curve and lower rates of complications compared to AUS placement.^17^ Furthermore, compared to AUS, the male sling does not require manual operation by the patient, which is a common reason why a significant amount of men would prefer a sling compared to an AUS.^18^ Despite the increased uptake of slings over the 23‐year study period, year‐on‐year analyses reveal that since FY2011/12, male sling insertions have plateaued compared to AUS placement. This crescendo‐plateau pattern in the uptake of male slings is an observation that has been noted in the United States, the United Kingdom and Germany.^15^, ^19^, ^20^

It is unclear if this reduced enthusiasm for slings in recent years would be due to negative publicity from transvaginal slings. The recent MASTER trial may have initiated a shift in clinicians' preferences. This randomised non‐inferiority clinical trial demonstrated comparable continence rates between male slings and AUS; however, the post hoc revealed that the AMS 800 device outperformed male slings in nearly all secondary outcomes, including postoperative continence, patient satisfaction, and complication rates. Additionally, the MASTER trial did not evaluate the anticipated advantages of male slings, such as shorter hospital stays and lower costs, over the AMS 800 device.^21^ Furthermore, the evolving availability of sling systems may have contributed to the stabilisation of male slings noted in our analysis. Several sling systems, such as Virtue, have been withdrawn from the Australian market, whereas the adoption of newer adjustable devices (such as ATOMS, ARGUS) is primarily used by only a handful of surgeons nationwide (Table S1).

In our study, AUS procedures grew by 458% demonstrating their widespread acceptance and utility in managing male stress incontinence in Australia. By FY2022/23, AUS procedures accounted for 58% of all interventions firmly establishing their role as the standard of care for patients, predominantly those aged 70–74 years. The parallel rise in AUS revision procedures, increasing by 444%, aligns with the known challenges of maintaining long‐term device functionality, which often necessitates replacements or revisions. Reported revision rates range from 26% to 50.8%, and long‐term studies have shown that only 5% of devices remain revision‐free at 20 years.^22^, ^23^ AUS failure is most often due to mechanical malfunction, urethral atrophy, erosion or infection, with reported rates varying widely across studies.^24^ The marked increase in revision rates across the study period may also be due to improved prostate cancer survivorship. As patients live longer after radical prostatectomy or radiation, a larger proportion of men outlive the durability of their implant, hence necessitating revision or replacement. Nevertheless, this underscores the importance of patient selection and counselling when considering AUS. Factors such as patient age, overall health, manual dexterity and presence of comorbidities must be carefully considered.

Conversely, the use of paraurethral bulking agent procedures decreased 62.5% over the same period, highlighting a declining role in clinical practice. This declining trend was also observed in the United States and European populations.^25^ This overall move away from bulking agents is likely due to the poor short‐term efficacy of this treatment. Imamoglu et al. compared the efficacy of paraurethral bulking agents versus AUS and reported a significant difference in continence rates favouring AUS implantation (72% for AUS versus 23% for paraurethral bulking agents).^26^ However, their overall efficacy is less favourable compared to slings. Sacco et al. conducted a propensity score–matched analysis comparing bulking agents to the TiLOOP transobturator male sling. This study found failure‐free rates of 15% and 77%, respectively, with satisfaction rates of 3.8% versus 71%. Additionally, bulking agents have poor durability in results, with patients often requiring repeat injections after 6 months.^27^ In our data set, over the past 10 years, paraurethral bulking agents have been utilised predominantly in older age groups (70–74 year of age), compared to the other surgical treatments available.

The escalating costs of male stress incontinence management reflect a combination of increasing procedural volumes, rising complexity and technological advancements. The cost analysis further emphasises the growing financial burden of male stress incontinence management. Total expenses in our study escalated by 555.7% over the study period, with AUS procedures contributing disproportionately. Insertion costs alone rose by 913%, while revision and replacement procedures saw a 616% increase over the study period. This trend is consistent with international data, where AUS procedures are amongst the costliest. For instance, in the United States, mean total costs per AUS procedures range from $72 887 to $92 045.^28^ European countries with government‐subsidised healthcare report lower but still substantial expenses, underscoring the universal challenge of affordability in advanced surgical care.^29^ Sling procedures, despite their significant volume growth, showed a more modest cost increase, reflecting their relatively lower technical and resource requirements. Meanwhile, paraurethral injections maintained stable costs, aligning with reduced utilisation and lower procedural complexity.

State‐level analysis highlights regional disparities in procedural costs and utilisation. New South Wales consistently contributed the highest to the national aggregate cost, reflecting its prominence in procedural volumes. Other states exhibited variable trends, such as cost reductions in sling procedures in Queensland and South Australia, contrasting with cost increases in New South Wales and Victoria. These variations may stem from differences in healthcare infrastructure, access and local clinical practices. Comparable patterns are observed internationally, where urban centres in high‐income countries typically demonstrate higher procedural volumes and costs due to advanced healthcare facilities and greater adoption of innovative techniques.^30^ Conversely, states like Tasmania and Northern territories reported minimal procedural growth and, in some cases, cost reductions, potentially signalling limited access to advanced interventions.

A key strength of this study is the incorporation of data from the AIHW, which encompasses both public and private hospitals. This 23‐year longitudinal study is the first analysis of surgical trends of male SUI within an Australian setting. Nevertheless, it is important to acknowledge some limitations of the study. First, this study only includes procedural codes available on AIHW; there are several new MBS codes related to sling procedures that are not currently registered under the AIHW male SUI procedural group (e.g., 37 040, 37 042, 37 341 and 37 344; see Table S2); hence, our findings may underestimate synthetic and autologous slings usage in Australia. Importantly, revision codes also cannot distinguish revisions procedures, from explantation followed by new device implantation. Despite these limitations, our study should encourage practitioners to appropriately utilise these new procedural codes to improve the accountability and representation of various male SUI procedures available through MBS. Future studies should evaluate trends following the incorporation of these new procedural codes into AIHW and for comparison of different SUI products to assess market trends. Lastly, this is an observational study so it would be difficult to establish any causal relationships from our findings and only speculations can be made.

## 5 CONCLUSION

In summary, this 23‐year national analysis demonstrates a significant evolution in the surgical management of male stress urinary incontinence within Australia. The marked increase in AUS implantation, alongside a concurrent rise in revision rates, reflects both improved accessibility, procedural expansion and the inherent long‐term challenges of prosthetic continence devices. Male slings have become an established adjunct, though recent data suggest stabilisation in their uptake. This may be due to improved surgeon familiarity with AUS or patient‐perceived harms of slings. Contrastingly, paraurethral bulking agents have shown a steady decline, consistent with their limited efficacy and durability in male cohorts.

## AUTHOR CONTRIBUTIONS

*Conceptualization*: Yam Ting Ho, Omattage Mahasha Perera. *Methodology*: Yam Ting Ho, Omattage Mahasha Perera, Femi E. Ayeni. *Formal analysis*: Yam Ting Ho, Omattage Mahasha Perera. *Data curation*: Yam Ting Ho, Omattage Mahasha Perera. *Writing—original draft preparation*: Yam Ting Ho, Omattage Mahasha Perera. *Writing—review and editing*: Yam Ting Ho, Omattage Mahasha Perera, Femi E. Ayeni. *Visualisation*: Yam Ting Ho, Omattage Mahasha Perera. *Resource*: Femi E. Ayeni. *Validation*: Eric Chung, Vincent Tse, Devang Desai. *Supervision*: Eric Chung, Vincent Tse, Devang Desai. All authors have read and agreed to the published version of the manuscript.

## CONFLICT OF INTEREST STATEMENT

There are no conflicting interests to declare.

## Supporting information


**Figure S1.** YoY change for each individual type of male SUI procedure between FY2000/01–2022/23. Only exception is retropubic procedure starts from FY2004/05. 4images above
**Figure S2.** YoY change for revision procedures between FY2000/01–2022/23. Three images above
**Figure S3.** Inner ring is 2002/03, middle ring is 2012/13 and outer ring is 2022/23
**Figure S4.** Age distribution for all SUI procedures
**Figure S5.** Age distribution for all revisions
**Table S1.** MBS codes that are currently not available on AIHW
**Table S2.** Male slings available
