# Supplementary material for: Male stress urinary incontinence surgery in Australia: Temporal trends over two decades
Source: BJUI Compass. 2026 Apr 22;7(4):e70205. doi: 10.1002/bco2.70205 (PMC13100885; doi:10.1002/bco2.70205)
Supplement: Supplementary file 1 — Figure S1. YoY change for each individual type of male SUI procedure between FY2000/01–2022/23. Only exception is retropubic procedure starts from FY2004/05. 4images above Figure S2. YoY change for revision procedures between FY2000/01–2022/23. Three images above Figure S3. Inner ring is 2002/03, middle ring is 2012/13 and outer ring is 2022/23 Figure S4. Age distribution for all SUI procedures Figure S5. Age distribution for all revisions Table S1. MBS codes that are currently not available on AIHW Table S2. Male slings available [file BCO2-7-e70205-s001.docx]

**Supplementary material**

Figure S1 – YoY change for each individual type of male SUI procedure between FY2000/01-2022/23. Only exception is retropubic procedure starts from FY2004/05. 4images above

Figure S2 – YoY change for revision procedures between FY2000/01-2022/23. Three images above

Figure S3 - Inner ring is 2002/03, middle ring is 2012/13 and outer ring is 2022/23

Figure S4 - Age distribution for all SUI procedures

 Figure S5 – Age distribution for all revisions

Table S1. MBS codes that are currently not available on AIHW

| Code |  |
| --- | --- |
| 37040 | Bladder stress incontinence, sling procedure for, using a non-adjustable synthetic male sling system |
| 37042 | Bladder stress incontinence—sling procedure for, using autologous fascial sling |
| 37341 | Urethral sling, division or removal of, suprapubic, combined suprapubic and vaginal or combined suprapubic and perineal approach other than a service associated with a service to which item 37340 or 37344 applies |
| 37344 | Urethral autologous fascial sling (or other biological sling), division or removal of, other than a service to which 37340 or 37341 applies |

Table S2. Male slings available

| Name | Sponsor | Features |
| --- | --- | --- |
| InVance | American Medical Systems | Bone-anchored sling, Non-adjustable |
| AdVance | American Medical Systems | Transobturator sling, Non-adjustable |
| Argus | Endotherapeutics Pty Ltd | Retropubic sling, Adjustable |
| Remeex | Gytech Pty Ltd | Retropubic sling, Adjustable |
| Virtue | Coloplast Pty Ltd | Quadratic sling (Transobturator and pre-pubic), Non-Adjustable |
| TiLOOP | Medical Specialties Australia Pty Ltd | Transobturator sling, Non-adjustable |
